# Overcoming Cabbage Crossing Incompatibility by the Development and Application of Self-Compatibility-QTL- Specific Markers and Genome-Wide Background Analysis

**DOI:** 10.3389/fpls.2019.00189

**Published:** 2019-02-26

**Authors:** Zhiliang Xiao, Fengqing Han, Yang Hu, Yuqian Xue, Zhiyuan Fang, Limei Yang, Yangyong Zhang, Yumei Liu, Zhansheng Li, Yong Wang, Mu Zhuang, Honghao Lv

**Affiliations:** Institute of Vegetables and Flowers, Chinese Academy of Agricultural Sciences, Key Laboratory of Biology and Genetic Improvement of Horticultural Crops, Ministry of Agriculture, Beijing, China

**Keywords:** cabbage, crossing incompatibility, quantitative trait loci, genetic mapping, genomic background analysis

## Abstract

Cabbage hybrids, which clearly present heterosis vigor, are widely used in agricultural production. We compared two *S5* haplotype (Class II) cabbage inbred-lines 87–534 and 94–182: the former is highly SC while the latter is highly SI; sequence analysis of SI-related genes including *SCR*, *SRK*, *ARC1*, *THL1*, and *MLPK* indicates the some SNPs in *ARC1* and *SRK* of 87–534; semi-quantitative analysis indicated that the SI-related genes were transcribed normally from DNA to mRNA. To unravel the genetic basis of SC, we performed whole-genome mapping of the quantitative trait loci (QTLs) governing self-compatibility using an F_2_ population derived from 87–534 × 96–100. Eight QTLs were detected, and high contribution rates (CRs) were observed for three QTLs: *qSC7.2* (54.8%), *qSC9.1* (14.1%) and *qSC5.1* (11.2%). 06–88 (CB201 × 96–100) yielded an excellent hybrid. However, F_1_ seeds cannot be produced at the anthesis stage because the parents share the same *S-*haplotype (*S57*, class I). To overcome crossing incompatibility, we performed rapid introgression of the self-compatibility trait from 87–534 to 96–100 using two self-compatibility-QTL-specific markers, BoID0709 and BoID0992, as well as 36 genome-wide markers that were evenly distributed along nine chromosomes for background analysis in recurrent back-crossing (BC). The transfer process showed that the proportion of recurrent parent genome (PRPG) in BC_4_F_1_ was greater than 94%, and the ratio of individual SC plants in BC_4_F_1_ reached 100%. The newly created line, which was designated SC96–100 and exhibited both agronomic traits that were similar to those of 96–100 and a compatibility index (CI) greater than 5.0, was successfully used in the production of the commercial hybrid 06–88. The study herein provides new insight into the genetic basis of self-compatibility in cabbage and facilitates cabbage breeding using SC lines in the male-sterile (MS) system.

## Introduction

Cabbage (*Brassica oleracea* L. var. *capitata*), a cole crop species, is a vegetable of worldwide economic importance due to its strong resistance, wide adaptability, favorable taste and healthcare-related value (Fang, 2000). Cabbage hybrids, which clearly present heterosis vigor, are widely used in cabbage production, and self-incompatible (SI) lines and male-sterile (MS) lines are two important tools for utilizing cabbage heterosis. Since the 1970s, attention has been paid to the selection of SI lines for the seed production process. However, self-incompatibility-based hybrid seed production is labor intensive, as the two parental lines can be reproduced only by artificial pollination; moreover, the purity of hybrid seeds never reaches 100%. Since the late 1990s, breeders have been turning to applying cytoplasmic male-sterile (CMS) or dominant genetic male-sterile (DGMS) lines for cabbage hybrid seed production (Pelletier et al., 1983; Earle et al., 1994; Fang et al., 1997), and many SI lines are still used in the MS system.

Self-incompatibility, the recognition and rejection of self-pollen, is a widespread mechanism by which flowering plants prevent self-fertilization. Pollen genotypes are determined by diploid pollen wall proteins: when the stigma and pollen have the same *S*-haplotype, they will present an incompatible reaction. This system retains genetic variation and avoids inbreeding depression in more than half of angiosperm species. Cabbage sporophytic SI is regulated by a single multi-allelic *S*-locus (Bateman, 1954; Thompson, 1957), which consists of the following three pollen–stigma recognition-related genes: *SLG* (*S*-locus glycoprotein), *SRK* (*S*-locus receptor kinase), and *SCR*/*SP11* (*S*-locus cysteine-rich protein and *S*-locus protein 11) (Nasrallah, 1974; Stein and Nasrallah, 1993; Kusaba et al., 2000; Takasaki et al., 2000; Watanabe et al., 2000; Nettancourt, 2001; Takayama et al., 2001; Takayama and Isogai, 2005; Fujimoto et al., 2006). The specific interaction between *SRK*, which is the female determinant expressed in stigmas, and *SCR*/*SP11*, which is the male determinant expressed in pollen grains, induces a signaling cascade within stigma epidermal cells. This signaling network provides stigmas with the ability to recognize and reject self-pollen grains. In addition, *ARC1* (arm repeat containing 1), *THL1* (thioredoxin-h-1), and *MLPK* (M-locus protein kinase), which are not associated with the *S* locus, are also involved in pathways related to pollen–stigma interactions. *THL1* is a negative regulator (Cabrillac et al., 2001; Haffani et al., 2004), while *ARC1* and *MLPK* are positive regulators (Stone et al., 1999, 2003; Murase et al., 2004). As a relative characteristic of self-incompatibility, self-compatibility has also been analyzed in Brassica crops, including *B. oleracea, B. rapa*, and *B. napus* (Okuda et al., 2000; Murase et al., 2004; Okamoto et al., 2007; Boggs et al., 2009). Some studies have shown that genetic variation in *S*-locus genes may be responsible for self-compatibility, such as *SCR* (Okamoto et al., 2007), *SRK* (Gaude et al., 1993; Isokawa et al., 2010), *THL1* (Bower et al., 1996), *ARC1* (Stone et al., 1999), *MLKP* (Murase et al., 2004; Hatakeyama et al., 2010). In addition, Kakita et al. (2007) and Isokawa et al. (2010) reported self-compatibility traits that are inconsistent with known *S*-loci and concluded that new loci may lead to self-compatibility.

Self-compatible (SC) lines facilitate cabbage hybrid seed production by two aspects. (i) These lines reduce costs during the parental line reproduction process; the male parental line and the maintainer line can be made to be SC lines, whose reproduction can be performed by honeybee pollination. (ii) These lines avoid crossing incompatibility during the hybrid seed production process, as parental lines with the same *S*-haplotype cannot produce hybrid seed by honeybee pollination. Therefore, the development of SC lines is urgently needed to meet the needs of improvements in cabbage breeding. Approximately 50 forms have been characterized in cabbage (Bateman, 1952; Thompson, 1957; Ockendon, 2000). Based on the sequence similarities between *SLG* and *SRK*, the *S* haplotypes are categorized as Class I or Class II. In *B. oleracea*, only three Class II *S* haplotypes have been identified (i.e., *S2*, *S5*, and *S15*). Class I *S* haplotypes are generally dominant over Class II *S* haplotypes. Tian et al. (2013) identified 26 *S*-haplotypes in cabbage, e.g., *S2*, *S5*, and *S15*, which can provide a basis for quick analyses of *S*-haplotypes in cabbage. Stern et al. (1982) and Fang et al. (1983) proposed methods of compatibility index (CI) calculations and fluorescence microscopy observations to assess the self-compatibility phenotype of cabbage; these methods are widely used in compatibility identification.

In the current study, we determined that 87–534 was an elite cabbage line that carried the *S5* haplotype (Class II) with a high compatibility index (CI; i.e., >10.0). 94–182 consisted of the same haplotype, but had a very low CI value (<1.0). We conducted sequence and expression analyses of the SI-related genes in 87–534 and 94–182 plants to clarify why 87–534 was highly self-compatible, and mapped the quantitative trait loci (QTLs) associated with self-compatibility using a segregating population derived from 87–534 × 96–100. We also applied genome-wide background markers and self-compatibility-QTL-specific markers in recurrent back-crossing (BC) for rapid introgression of the self-compatibility trait from 87–534 to 96–100; and used the newly developed line SC96–100 to disrupt the crossing incompatibility and generate an excellent hybrid similar to 06–88. The study here provides new insight into the genetic basis of self-compatibility in cabbage and facilitates cabbage breeding via SC lines in the MS system.

## Materials and Methods

### Plant Materials

**FIGURE 1:**
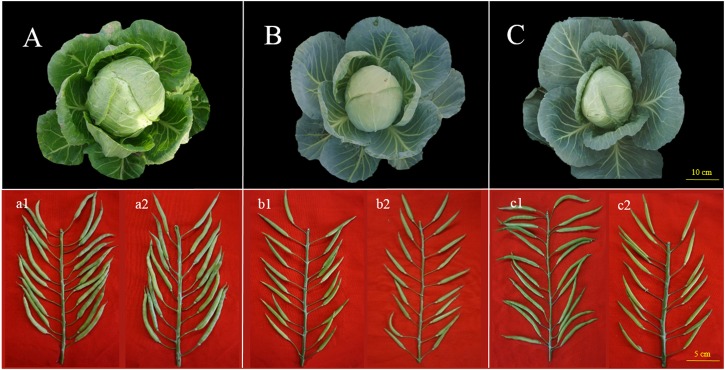
Field performance and fruiting performance of 87–534, 96–100, and 06–88. **(A–C)** Field performance of 87–534, 96–100, and 06–88, respectively; **(a1,b1,c1)**: seed setting from self-pollination at the anthesis stage of 87–534, 96–100, and 06–88, respectively; **(a2,b2,c2)**: seed setting from self-pollination at the bud stage of 87–534, 96–100, and 06–88, respectively.

Lines 87–534 is an elite line originating from the cultivar ‘Flstacus’ and introduced from Germany by IVF-CAAS in 1987; its CI is greater than 10.0 at the anthesis stage, and its *S*-haplotype is *S5* (class II), as identified in our previous study (Tian et al., 2013). The field and podding performance of this line is shown in Figure 1A (a1 and a2).

Lines 94–182, introduced to China from United States by IVF–CAAS in 1994, is a cabbage inbred line with a CI value < 1.0. Its *S* haplotype is also *S5* (Class II).

Line 96–100, the *S*-haplotype of 96–100 is *S57* (class I) (Tian et al., 2013), and the CI (number of seeds per pod) at the anthesis stage < 1.0. The field and podding performance is shown in Figure 1B (b1 and b2). Lines 96–100 is a parent of several excellent cabbage hybrids, including Zhonggan 18, Zhonggan 828, and Zhonggan 588 (Yang et al., 2004; Zhang et al., 2014).

Line CB201 is an elite line derived from the cultivar ‘CB201’ and introduced from Thailand by IVF-CAAS in 2002; its *S*-haplotype is *S5* (class II).

Hybrid 06–88 is an elite F_1_ derived from CB201 × 96–100 (Figure 1C). However, F_1_ seeds cannot be produced via pollination at the flower stage due to the same haplotype; however, the CI is normal at the bud stage [Figure 1 (c1 and c2)].

To study the genetic basis of self-compatibility by QTL mapping and transfer the self-compatibility trait to 96–100, the highly SC line 87–534 was crossed with 96–100 to produce an F_1_ population, from which an F_2_ population comprising 230 individuals was obtained. Each F_2_ individual was self-pollinated at the anthesis stage, and the CI was calculated at the podding stage.

### Phenotyping

The self-compatibility phenotype was assessed based on the CI and fluorescence microscopy. The CI was determined using a published procedure (Fang et al., 1983). Three individuals were manually self-pollinated (approximately 10 flowers per individual were selected) 1 or 2 days after the flowers had fully opened. The CI was then calculated at the podding stage via the following formula: CI = number of seeds/number of pollinated flowers. The self-incompatibility/self-compatibility levels were rated as follows: self-incompatibility: CI < 1; moderate self-compatibility (MSC): 4 ≤ CI ≤ 1; self-compatibility: CI > 4.

Cabbage samples were also analyzed by fluorescence microscopy as previously described (Stern et al., 1982). For each individual, five flowers were manually self-pollinated 1 or 2 days after they had fully opened. The stigmas were harvested 24 h later and fixed in formalin–acetic acid–alcohol fixation fluid for 24 h, following preservation in 70% ethanol. The samples were treated with 1 M NaOH, incubated in a water bath at 60°C for 1 h, washed three times with distilled water, and then stained with 0.1% aniline blue solution (0.1 M K_3_PO_4_ and 0.1% aniline blue) for 12 h. A fluorescence microscope was used to count the number of pollen tubes that had penetrated the stigmas (NPT) (Martin, 1959). The self-incompatibility levels were rated as follows: self-incompatibility: NPT < 10; MSC: 10 ≤ NPT ≤ 25; self-compatibility: NPT > 25.

Three independent repetitions were performed to obtain a mean value. All pollinations were conducted in the experimental greenhouse of IVF-CAAS, Beijing, China, between 9:00 and 10:00 a.m. on sunny days in late April at 20–25°C to avoid bad weather conditions inappropriate for pollination.

### Polymerase Chain Reaction Amplification and Expression Analysis of Self-Incompatibility–Related Genes

Total RNA was extracted from the stigmas and anthers of 87–534 and 94–182 plants using the EasyPure^TM^ Plant RNA Kit (TransBionovo Co., Beijing, China). The cDNA templates for all genes, except for *SCR*, were prepared using RNA extracted from anthers. The purified RNA was reverse transcribed to cDNA using the EasyScript^TM^ Reverse Transcriptase Kit (TransBionovo). For polymerase chain reaction (PCR) amplifications and semi-quantitative analysis of SI-related genes, specific primers (Supplementary Table S1) were designed to amplify whole coding sequences. The reference sequences of *S-*related genes were obtained from the NCBI [*SRK* (*AB024416.1*), *SCR* (AB067448), *ARC1* (EU344909), *MLPK* (AB121973), *THL1* (AF273844.1)], we also blasted these genes on the *Brassica* genome ‘02-12’^1^ (Liu et al., 2014) and ‘TO1000’ genome^2^ (Parkin et al., 2014) to obtain more information. The primers were designed by Premier (Version 5.0^3^). The actin 1 gene served as the reference gene (Zhao et al., 2012). The PCR products were separated by 1% agarose gel electrophoresis (130 V). The target DNA bands were purified using the EasyPure^TM^ Quick Gel Extraction Kit (TransBionovo) and sequenced. Three technical replicates were performed for each gene. The relative changes in gene expression levels were calculated using the 2^-ΔΔCT^ method (Livak and Schmittgen, 2001).

### Molecular Marker Design and Genotyping

Total genomic DNA was extracted from the young leaves of all individuals using cetyltrimethylammonium bromide according to a published method (Murray and Thompson, 1980). The DNA concentrations were determined using a NanoDrop ND-100 spectrophotometer (Thermo Fisher Scientific Co., Wilmington, DE, United States) and then diluted to a working concentration of 40–50 ng/μl for subsequent PCR.

To design primers for insertion–deletion (InDel) markers, whole-genome resequencing (approximately 10× coverage) of the parental lines (i.e., 87–534 and 96–100) was performed. Totals of 7.2 and 7.1 Gb of Illumina paired-end reads were generated for 87–534 and 96–100, respectively. The *B. oleracea* ‘02-12’ reference sequence was retrieved from ‘BRAD’ for resequencing data alignment and for detecting sequence polymorphisms between the parental lines (Cheng et al., 2011; Liu et al., 2013; Lv et al., 2014a). To avoid the detection of false polymorphisms, multi-hit reads were filtered and removed from the dataset, and only single-hit reads were used to design primers. All the primers used were designed in accordance with the following parameters: amplicon length, 100–200 bp; primer length, 19–25 bp; differential fragment length, 3–6 bp; and melting temperature, 53–58°C. A total of 2000 primer pairs for the polymorphic InDel markers were designed, and 1000 pairs that were evenly distributed across nine chromosomes were selected for further analyses. These InDel primers were used for whole-genome genomic background analyses in 87–534 and 96–100 to identify the polymorphic markers on different chromosomal segments. Individual F_2_ plants were then screened via the polymorphic markers.

Each 10 μl PCR mixture contained 1 μl of PCR buffer (10×, Mg^2+^ included), 0.8 μl of dNTPs (2.5 mM each), 0.2 μl of Taq DNA polymerase (2.5 U/μl), 2.5 μl of DNA template (40 ng/μl), 0.3 μl of each forward and reverse primer (10 μM), and 9.8 μl of ddH_2_O. The reaction mixture was incubated in a GeneAmp PCR system 9700 (Applied Biosystems Inc., Foster City, CA, United States), and the PCR profile was as follows: initial 5 min at 94°C; 35 cycles of 30 s of DNA denaturation at 94°C, 30 s of annealing at 55°C and 45 s of extension at 72°C; and a final extension of 7 min at 72°C. With respect to polyacrylamide gel electrophoresis (PAGE), the PCR products were separated on 8% (w/v) polyacrylamide gels at 160 V for 1.5 h and then visualized with silver staining.

For each marker, individuals with the 87–534 allele were categorized as ‘a’. Individuals with the 96–100 allele were categorized as ‘b,’ and those with the F1 allele were categorized as ‘h.’

### QTL Mapping of Self-Compatibility

A linkage map was constructed using the Join Map 4.0 program with a minimum logarithm of odds (LOD) score of 4.0 (Van Ooijen, 2006). The Kosambi function was used to convert the recombinant value to genetic distance (Kosambi, 1944). A χ^2^ test for goodness of fit to the expected 1:1 Mendelian segregation ratio was performed to identify significantly skewed markers (*P* < 0.01).

Quantitative trait loci analysis was performed using QTL IciMapping version 4.0 (Meng et al., 2015) and QTL Cartographer version 1.13 (Basten et al., 2004). A forward–backward stepwise regression was performed to choose co-factors before performing QTL detection. A permutation test was performed with QTL Cartographer to estimate the appropriate significance threshold for analysis. A LOD threshold of 3.0, which corresponded to a genome-wide significance level of 0.10, was chosen. The resulting QTL names consisted of an abbreviated trait name followed by the chromosome and QTL codes. For example, *qSC4.1* represents the first QTL on chromosome 4 for self-compatibility.

### Development of Self-Compatibility Marker Combinations and Genomic Background Markers

The strategy of developing self-compatibility marker combinations involved both the selection of markers closely associated with the high contribution rate (CR) QTL trait of self-compatibility and the development of markers or marker combinations for screening individual F_2_ plants. Based on the CI, the best marker or marker combination was then used to identify the self-compatibility phenotype and applied to marker-assisted recurrent BC.

The genomic background analysis of the back-cross populations revealed that some of the polymorphic markers were evenly distributed across the polymorphic region in both 87–534 and 96–100.

### Marker-Assisted Recurrent Backcrossing

The donor parent 87–534 was crossed with the recipient parent 96–100 to obtain F_1_-generation plants, which were subsequently successively back-crossed with 96–100 to obtain back-cross populations. The best individuals of every population (200 individuals) were selected for further experiments; the genomic DNA was extracted from all individuals and subsequently analyzed with self-compatibility marker combinations, and the individual SC plants were saved. All the individual SC plants were subjected to genomic background analyses via background markers. The plants were phenotyped to characterize the overall performance of various plant traits. The main agronomic traits of back-cross individuals were examined, with 87–534 and 96–100 plants serving as reference materials. The individual plants that were phenotypically similar to 96–100 were transplanted to the greenhouse and subjected to vernalization. Based on CI and fluorescence microscopy, the best individual SC plant was selected for further BC. Finally, individuals that were highly SC with almost the same genetic background as that of the 96–100 plants were self-pollinated to generate materials that were homozygous for self-compatibility, which were named SC96–100 lines. SC96–100 was crossed with CB201 to test the transfer results (the self-incompatibility/self-compatibility ratio and the self-compatibility based on 06–88).

To further test the performance of SC96–100, the agronomic traits (head weight, length, width, and core length) of SC96–100 and SC06–88 were evaluated and compared with those of 96–100 and 06–88, respectively, according to the methods described in Lv et al. (2017).

## Results

### Phenotyping

**FIGURE 2:**
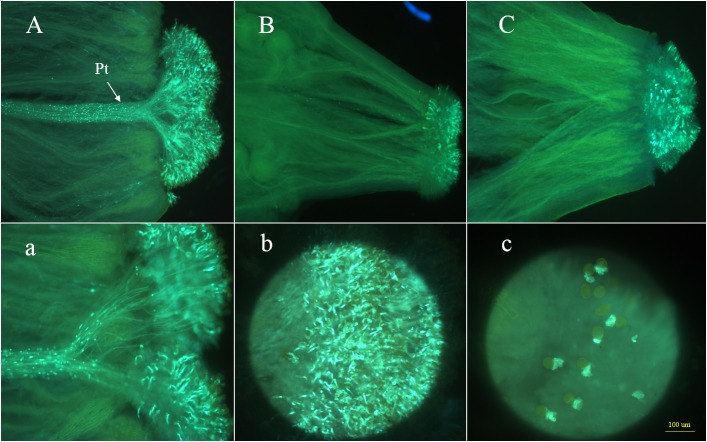
Observations of pollen tube germination at the stigmas of 87–534, 96–100, and 06–88. Panels **(A,a)** show the side views of the stigma of 87–534; Pt indicates the pollen tubes; panels **(B,C)** show the side views of the stigma of 96–100 and 06–88, respectively; panels **(b,c)** show the front views of the stigma of 87–534 and 96–100, respectively.

The CI values at the anthesis stage for 87–534 and 96–100 were 13.2 and 0.6, respectively. Additionally, microscopy analyses of the 87–534 samples revealed that an excess of 25 pollen tubes clustered together, germinated, and penetrated the stigmas (Figure 2A,a). In contrast, in the 96–100 samples, most pollen tubes failed to penetrate the stigmas. We observed that callose was deposited on the stigma surface and observed malformed pollen tubes that failed to grow (Figure 2B,b,c). The CI and microscopy results indicated that the 87–534 and 96–100 plants exhibited completely different SI phenotypes (i.e., the 87–534 plants were highly SC, while the 96–100 plants were highly SI). With respect to the hybrid 06–88, the CI value was 0.1, and microscopy analysis revealed that its pollen tubes also failed to penetrate the stigmas (Figure 2C). The results obtained via CI values and microscopy analysis are consistent, which was the same case in the study by Zhao (2007). For convenience, the CI value was used as the main evaluation criterion in this study.

### Analysis of the SI–Related Genes in Lines 87–534 and 94–182

Whole coding sequences of SI-related genes from 87–534 to 94–182 plants were amplified using gene-specific primers (Supplementary Table S1). The amplified sequences were aligned using the DNAMAN program (version 7.0)^4^. There were no differences between the *SCR*, *MLPK*, and *THL1* fragments of 87–534 and 94–182 plants. The *ARC1* sequence was also similar between 87–534 and 94–182 plants (97.07% similarity), with some single nucleotide polymorphisms (SNP) causing 17 amino acid differences. For *SRK*, we detected three SNPs in the coding sequence and some SNPs in the intron region, however, these SNPs did not cause mutations in amino acids. The alignment of *ARC1* and *SRK* was shown at Supplementary Figures S1, S2.

The cDNA prepared using RNA extracted from 87–534 to 94–182 stigmas and anthers was subjected to semi-quantitative analysis, with an *Actin1* gene serving as a reference. For all SI-related genes (i.e., *SRK*, *SCR/SP11*, *THL1*, *MLPK*, and *ARC1*), we generated distinct DNA bands *via* PCR experiments (Supplementary Figure S3). This result indicated that the SI-related genes were transcribed normally from DNA to mRNA.

### Linkage Map Construction

Based on the whole-genome resequencing data (approximately 10× coverage) of the parental lines 87–534 and 96–100, we chose 1000 primer pairs as polymorphic InDel markers for nine chromosomes. We then selected 335 primer pairs that produced reliable PCR products to genotype the F_2_ mapping population. Of these markers, 302 were co-dominant, and 33 were dominant.

**FIGURE 3:**
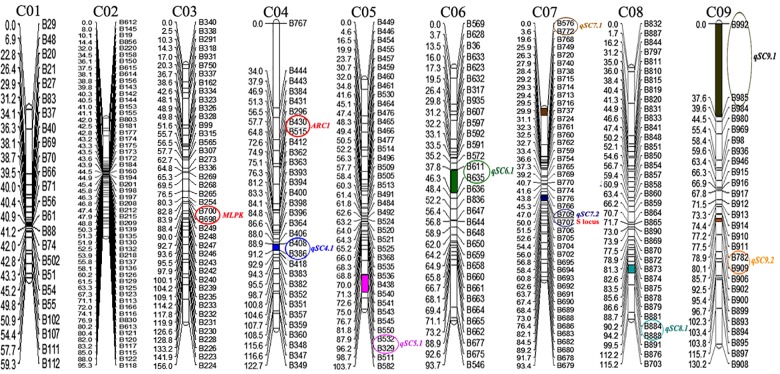
Genetic linkage map of cabbage. The genetic distance (cM) is indicated on the left side of linkage groups, and the marker number is indicated on the right side. The positions of self-compatibility QTLs in 87–534 were mapped onto the linkage map. The number at the top of each linkage group corresponds to the location of the genomic chromosomes in Brassica.

Join Map 4.0 software was used to construct nine linkage groups consisting of 329 markers (six markers did not map to any of the linkage groups) with a LOD threshold of 4.0 (Figure 3). The map spanned 969.5 cM, with an average marker interval of 2.95 cM. The linkage group lengths ranged from 59.3 to 156 cM, with 24–54 markers. The nine groups were anchored to their corresponding reference chromosomes (i.e., chromosomes C01–C09) according to the physical positions of the markers. The longest (156 cM) and shortest (59.3 cM) linkage groups were on chromosomes 3 and 1, respectively. The maximum (4.82 cM) and minimum (1.76 cM) average distances occurred on chromosomes 9 and 2, respectively. Chromosome 2 had the most markers (54), while chromosome 1 had the fewest (24). The largest interval between markers was 34.04 cM on chromosome 4 (between B767 and B444). Overall, the markers were relatively evenly distributed on the nine chromosomes (Table 1).

**Table 1:** Distribution characteristics of the genetic linkage map.

Chromosome	Length (cM)	No. markers	Average distance (cM)	Max gap (cM)	Distorted segregation
C01	59.3	24	2.47	15.89	3
C02	95.3	54	1.76	17.57	5
C03	156	41	3.80	16.35	7
C04	122.7	31	3.96	34.04	9
C05	103.7	39	2.66	11.94	5
C06	93.7	32	2.96	15.64	3
C07	93.4	44	2.12	16.01	2
C08	115.2	37	3.11	15.0	6
C09	130.2	27	4.82	37.56	3


A total of 43 skewed markers (31.9%) were detected based on a χ^2^ test for goodness of fit to the expected 1:1 Mendelian segregation ratio (*P* < 0.01). Twenty-four of the skewed markers were from the parent 96–100. Although the segregation ratio for some markers was considerably different from the expected 1:1 ratio, there was an adequate distribution of markers in the different linkage groups. These results were comparable to those of previous studies involving other mapping populations of *Brassica* crop species (Foisset et al., 1996; Voorrips et al., 1997; Lv et al., 2014b, 2016).

### Major QTLs Associated With Self-Incompatibility Were Identified

The self-compatibility phenotype was characterized using the CI evaluation method for each individual F_2_ plant; the CI values for each individual F_2_ plant are listed in Supplementary Table S2, and a frequency histogram was obtained, as shown in Supplementary Table S2. The CI exhibited a continuous and skewed distribution; more than 70% of the individuals had CI values lower than 1.0, indicating that the self-compatibility phenotype was controlled by major effect genes.

IciMapping software was used to perform QTL analysis for self-compatibility traits based on the constructed linkage groups and self-compatibility trait values. With a LOD threshold of 3.0, eight QTLs were detected: *qSC4.1*, *qSC5.1*, *qSC6.1*, *qSC7.1*, *qSC7.2*, *qSC8.1*, *qSC9.1*, and *qSC9.2.* Three high-CR loci were identified, including *qSC7.2* (54.81%), *qSC9.1* (14.14%), and *qSC5.1* (11.25%) (Table 2). *qSC7.2*, which presented the highest CR, was located at an interval on C07 harboring the *S*-locus, indicating that it might be a main effect locus conferring the self-compatibility phenotype to the plants. Additionally, there are higher-effect QTLs (*qSC9.1*, *qSC5.1*) that present a lower CR than did *qSC7.2*, indicating that other loci related to self-compatibility traits other than the *S*-locus exist. *qSC9.1* exhibited a negative additive effect for self-compatibility (the additive effect was -0.20).

**Table 2:** Quantitative trait loci (QTL) analysis of the self-compatibility trait in the F_2_ population.

QTL^a^	Chr^b^	LOD^c^	Position (cM)^d^	Marker interval^e^	Nearest marker^f^	Distance from the nearest (cM)^g^	*R*^2^ (%)^h^	Additive effect^i^	Dominant effect^j^
*qSC*4.1	C04	3.35	91	B408-B386	B386	0.21	2.55	0.96	–0.23
***qSC*5.1^k^**	**C05**	**7.06**	**96**	**B532-B329**	**B329**	**0.19**	**11.25**	**0.41**	**8.00**
*qSC*6.1	C06	4.45	46	B611-B635	B635	0.26	3.42	1.18	–0.28
*qSC*7.1	C07	3.81	1	B576-B772	B576	1.00	9.36	0.92	3.61
***qSC*7.2**	**C07**	**45.94**	**48**	**B707-B709**	**B709**	**1.11**	**54.81**	**3.90**	–**3.37**
*qSC*8.1	C08	3.61	92	B884-B888	B884	1.84	3.04	–1.07	–0.10
***qSC*9.1**	**C09**	**5.21**	**2**	**B985-B992**	**B992**	**5.21**	**14.14**	–**0.20**	**6.54**
*qSC*9.2	C09	3.18	79	B782-B909	B782	0.15	2.41	–0.95	–0.12


*^a^Name of the QTL. ^b^Position of the peak marker or marker interval. ^c^Peak marker or marker interval. ^d^Peak marker or marker interval. ^e^Peak marker or marker interval. ^f^Peak marker or marker interval. ^g^Peak marker or marker interval. ^h^Peak marker or marker interval. ^i^Additive effect: a positive additivity indicated that 96-100-308 carries the allele for an increase in the trait value, while a negative additivity means that 01-20 carries the allele for an increase in the trait value. ^j^Proportion of phenotypic variance explained by each QTL. ^k^Robust QTLs are indicated in bold.*

*qSC7.1*, *qSC4.1*, *qSC6.1*, *qSC8.1* and *qSC9.2* had low CRs of 9.36, 2.55, 3.42, 3.04, and 2.41%, respectively.

### Self-Compatibility-Specific Markers and the Development of Background Markers

Three QTLs (*qSC7.2, qSC5.1*, and *qSC9.1*) with high CRs were obtained by QTL mapping; these QTLs’ linkage markers (BoID0709, BoID0329, and BoID0992) were selected to develop self-compatibility marker combinations. The marker combinations were as follows: BoID0709, BoID0992 + BoID0329, BoID0709 + BoID0992, and BoID0709 + BoID0329. In addition, *qSC7.2* and *qSC5.1* had a positive additive effect, while *qSC9.1* had a negative additive effect. Therefore, we screened individual plants that had the same allele as that in 87–534 for markers BoID0709 and BoID0329 and plants that had the same allele as that in 96–100 for marker BoID0992. According to the CI of the individual F_2_ plants, the accuracy of the identification of the self-compatibility marker combinations was analyzed.

The results showed that BoID0709 + BoID0992 yielded the highest correctness (94.12%), followed by BoID0709 (74.24%), BoID0992 + BoID0329 (23.53%), and BoID0709 + BoID0329 (83.33%). Based on the data above, BoID0709 and BoID0992 were applied to marker-assisted selection (MAS) for self-compatibility. The primer sequences used are shown in Supplementary Table S3.

Based on the linkage map, 36 polymorphic markers that were evenly distributed on each chromosome were selected as background markers; the information on these markers is shown in Supplementary Table S3.

### Successful Introgression of Self-Compatibility From 87–534 to 96–100

For every back-cross population, individual plants that had the same allele as that in 87–534 for marker BoID0709 and plants that had the same allele as that in 96–100 for marker BoID0992 were chosen for further BC; the numbers of individuals that met these criteria in every population were 100 (F_1_), 102 (BC_1_), 98 (BC_2_), 99 (BC_3_), and 106 (BC_4_). The segregation ratio for both the F_1_ and BC populations conformed to a Mendelian ratio of 1:1, according to results of a χ^2^ test.

Thirty-six polymorphic markers were used to analyze the genetic backgrounds of these individual plants after the self-compatibility marker combinations were used. The recurrent parent genome (PRPG) of every population comprised the F_1_ (43.10–55.17%), BC_1_ (56.90–68.97%), BC_2_ (64.14–79.31%), BC_3_ (77.93–86.21%), and BC_4_ (86.55–93.10%), which indicated that the genetic background of the individual plants carrying the self-compatibility trait in every population gradually became similar to that of 96–100. We selected 30 individual plants that had a genetic background similar to that of the 96–100 plants for BC with 87–534 plants to generate BC_4_ plants whose backgrounds were also analyzed as described above.

**FIGURE 4:**
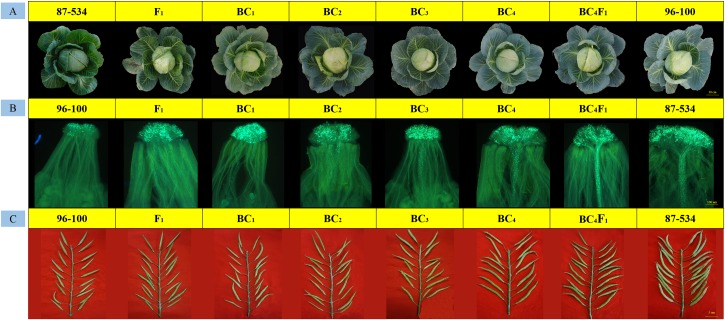
Field performance, fruiting performance and pollen tube germination at the stigmas for every typical back-cross plant, 87–534, and 96–100. **(A)** Field performance; **(B)** pollen tube germination at the stigmas; **(C)** fruiting performance.

Phenotypic observations were performed to characterize the overall performance of various plant traits. Half of the plants that were similar to the 96–100 plants were selected for transplantation to the greenhouse. In addition, the back-cross individuals were phenotypically somewhat similar to the 96–100 plants, and the phenotypes of typical individual plants of each population are shown in Figure 4A. Every 15 individual plants were self-pollinated at the anthesis stage, and fluorescence microscopy revealed that the number of pollen tubes gradually increased (Figure 4B).

At the seed-filling stage, we also observed that the seed setting of every population gradually increased (Figure 4C). The CI values were 0.17–1.10 (F_1_), 0.42–1.61 (BC_1_), 0.67–2.59 (BC_2_), 0.71–3.33 (BC_3_), and 2.30–4.71 (BC_4_) (Supplementary Table S4), which is consistent with the observed results. In addition, some BC_4_ individuals reached the self-compatibility level.

BC_4_F_1_ individuals were ultimately generated from self-pollinated progenies of the BC_4_ individuals. We also identified 200 BC_4_F_1_ individuals by self-compatibility-specific markers and obtained 195 individuals with self-compatibility traits. The CI and fluorescence microscopy analyses revealed that the BC_4_F_1_ individuals reached the self-compatibility level (the CI was 5.19–7.60). The background similarity between BC_4_F_1_ individuals and 96–100 individuals ranged from 95 to 97%, and the main agronomic characteristics of the former were generally the same as those of 96–100. We therefore named the BC_4_F_1_ plants as SC96–100; this newly developed SC96–100 line reached the requirements for self-compatibility and is currently being applied for the production of cabbage hybrids.

**FIGURE 5:**
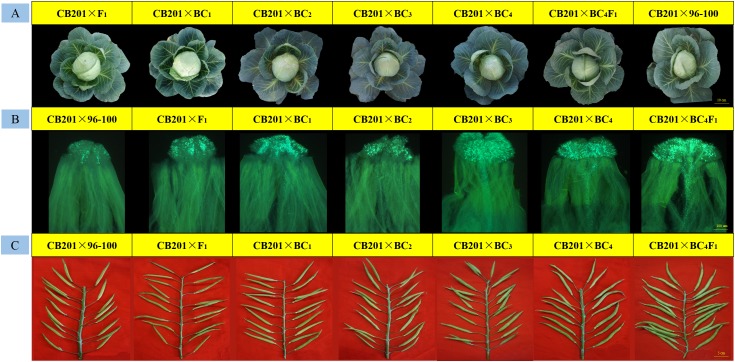
Field performance, fruiting performance and pollen tube germination at the stigmas for every typical BC × CB201 individual. **(A)** Field performance; **(B)** pollen tube germination at the stigmas; **(C)** fruiting performance.

Moreover, every back-cross population line was pollinated with CB201; the field performance, fluorescence microscopy and seed setting performance are shown in Figure 5. The results showed that the field performance of CB201 × SC96–100 was similar to that of CB201 × 96–100, and the number of pollen tubes and the CI also gradually increased. Furthermore, the CB201 × BC_4_F_1_ line reached the self-compatibility level as was named SC06–88, which can be applied for breeding.

The agronomic traits of 20 SC96–100 plants and 20 SC06–88 plants were compared with those of 96–100 plants and 06–88 plants, respectively, and the results are shown in Supplementary Table S5. Upon comparison, the agronomic traits of 96–100 and SC96–100 were, respectively, 0.7 kg/0.7 kg (head weight), 13.3 cm/12.9 cm (head length) and 5.3 cm/5.3 cm (core length); similarly, the agronomic traits of 06–88 and SC 06–88 were, respectively, 1.0 kg/1.1 kg (head weight), 13.7 cm/13.9 cm (head length) and 6.0 cm/5.9 cm (core length). The candidate agronomic traits of SC96–100 are similar to those of 96–100, and those of SC06–88 are also similar to those of 06–88. These results further showed that we successfully transferred self-compatibility from 87–534 to 96–100 and produced a new hybrid, SC06–88.

## Discussion

### Self-Incompatibility–Related Genes and Self-Compatibility Traits

The *SRK* and *SCR* genes are the female and male determinants, respectively, during pollen–stigma recognition in the SI process. Their interaction enables the stigma to recognize self-pollens. Mutations to either of the determinants may result in a shift from SI to SC, further confirming their separate functions (Goring et al., 1993; Schopfer et al., 1999; Takayama et al., 2000; Nasrallah et al., 2002; Ekuere et al., 2004). Nasrallah et al. (1992) observed that in a self-compatible *B. oleracea* mutant, the expression of *SRK* did not result in the recognition of self-pollen. However, pollen grains can still interact with the stigmas of plants with certain *S* haplotypes. Cabrillac et al. (1999) reported that the stigmas of plants with a mutated *SCR* were incapable of recognizing self-pollen, but still interacted with the pollen grains of specific *S* haplotypes. Nasrallah et al. (2002) showed that gene transfer of the stigma receptor kinase *SRK* and its pollen-borne ligand *SCR* from one *S-*locus haplotype of the SI and cross-fertilizing *Arabidopsis lyrata* is sufficient to impart SI phenotype in self-fertile *Arabidopsis thaliana*, which lacks functional orthologs of these genes. These results suggest that cross and reciprocal cross tests during the anthesis stage may enable researchers to deduce whether the stigmas and pollen grains are functionally normal (i.e., whether *SRK* and *SCR* are functional). Therefore, we analyzed 87–534 (highly self-compatible) and 94–182 (highly self-incompatible) plants, which carry the *S5* haplotype (Class II).

Previous studies revealed that in addition to *SRK* and *SCR*, some SI-related genes are important for normal SI functions (Boyes et al., 1991). For example, *THL1/THL2* is a negative regulator of the SI system that interacts with *SRK* and inhibits its activity in the absence of the *SCR* pollen protein (Bower et al., 1996; Cabrillac et al., 2001; Haffani et al., 2004). Additionally, *SLG* can enhance SI interactions, though it is not a required component (Gaude et al., 1993; Dixit et al., 2000; Takasaki et al., 2000). *ACR1* is a positive regulator of the SI system that can be activated by *SRK*, leading to the ubiquitination and degradation of stigma proteins, which ultimately results in pollen rejection (Stone et al., 1999, 2003). *MLPK*, which is another positive regulator, may form a complex with *SRK* to regulate upstream SI interactions (Murase et al., 2004; Kakita et al., 2007). Mutations to these SI-related genes may lead to SC. For example, *MLPK* belongs to the *RLCK* gene sub-family, and has eight conserved amino acids (G-G–G-V, A-K, E, DL-N, DFG, APE, D-WS-G, and R) (Steven and Hanks, 1991). Hatakeyama et al. (2010) constructed a detailed linkage map of *B. rapa* from the F_2_ progeny and Mapping of SI-related genes revealed that these QTL were co-localized with *SLG* on R07 and *MLPK* on R03. Murase et al. (2004) determined that Gly194 was replaced by Arg. Because Gly194 is a highly conserved amino acid in the protein kinase VIa sub-family, this mutation resulted in the loss-of-activity of *MLPK* and the development of the SC phenotype.

To confirm whether the SC traits in line 87–534 were caused by mutations in SI-related genes, we analyzed the sequences of these genes in 87–534 and 94–182 plants. Sequence analysis of SI-related genes including *SCR*, *SRK*, *ARC1*, *THL1*, and *MLPK* indicates some mutation in *SRK* and *ARC1*. We detect some mutations in the coding and conserved domains of the 87–534 genes. Semi-quantitative PCR results indicated these genes were normally expressed. These results imply that there are novel genetic factors associated with the SC phenotype of 87–534 plants.

### Quantitative Trait Locus Analysis of Self-Compatibility Traits

Except for the known SI-related genes, there are also other loci associated with SI/SC trait. Ma et al. (2009) mapped an *S* suppressor locus using a segregation population derived from S-1300 (SI) × 97-wen135 (SC) in *B. napus*. In Arabidopsis, Hülskamp et al. (1995) found the wax synthesis related genes *CER* was required for pollen-stigma recognition.

Till now, the mechanism of SC in *B. oleracea* has not been characterized. To unravel the novel genomic loci conferring SC traits to 87–534 plants, we conducted whole-genome QTL mapping experiments for the F_2_ population derived from the hybridization between 87–534 and 96–100 (highly self-incompatible; *S57* haplotype, Class I). Eight QTLs were detected on six chromosomes. No QTLs were detected on chromosomes 1–3. *qSC7.2* had the highest CR value, and was located in the same marker interval as *SRK* and *SCR* on chromosome 7, indicating that the *S*-locus contained the main-effect genes conferring the SC phenotype. However, QTLs were not associated with any of the SI-related genes, including QTLs with high CR values [i.e., *qSC9.1* (14.14%), *qSC7.1* (9.36%), and *qSC5.1* (7.06%)]. This observation suggests that there are novel genetic factors associated with SC traits. Additionally, the relationship between *S*-haplotypes and SC traits was consistent with the mapping results.

The candidate genes in the QTL regions were also discussed, and some of them might be good candidates, such as the genes involved in embryogenesis and pollen development. However, further work is still needed to fine-mapping and verify these genes, using a larger population. Besides, the markers used in this study may be useful for marker-assisted selection of self-compatible lines. In this study, the markers used in this study could be applied in MAS of SC lines; we used markers at both *qSC7.2* (BoID0709) and *qSC9.1* (BoID0992) to guarantee a high selection efficiency. Additionally, the results provide new insight into the mechanism of self-compatibility and can improve cabbage breeding via SC lines in the MS system.

### Powerful Breeding Method: Trait-Specific Markers Combined With Genomic Background Analyses

Marker-assisted selection is an important tool that is widely used in breeding and enables direct genotypic selection and effective gene polymerization via specific markers associated with target characteristics. In the study of resistance genes in cabbage (black rot, clubroot, and *Fusarium* wilt resistance, etc.), previous researchers have screened markers closely linked to these resistance genes and have applied them to cabbage breeding for disease resistance, which has provided important help for breeding resistant varieties (Landry et al., 1992; Pu et al., 2012; Kifuji et al., 2013). Genomic background analysis has also been applied to MAS to enable rapid and accurate breeding. Liu et al. (2017) and Yu et al. (2017) succeeded in transferring the *Fusarium* wilt resistance gene and the Ogu-CMS restorer gene by combining application of background selection and disease resistance-specific marker-assisted foreground selection.

In our study, we obtained self-compatibility-specific marker combinations for foreground selections and 36 genome-wide markers for background selections. Additionally, phenotypic observations (CI determination, fluorescence microscopy observation and agronomic traits) were also performed after our transfer process. A high selection efficiency in our transferring process indicated that MAS via multiple means will greatly improve the efficiency of breeding.

## Author Contributions

ZX wrote and revised the manuscript. ZX, FH, and YH isolated the samples and performed trait measurements, molecular experiments and marker assays. HL, YX, and MZ analyzed the trait and trial data and revised the manuscript. MZ, HL, and ZF conceived the idea and critically reviewed the manuscript. LY, YZ, YL, and ZL coordinated and designed the study. All authors read and approved the final manuscript.

## Conflict of Interest Statement

The authors declare that the research was conducted in the absence of any commercial or financial relationships that could be construed as a potential conflict of interest.

## Footnotes

1 http://brassicadb.org/brad/

2 http://plants.ensembl.org/Brassica_oleracea

3 http://www.premierbiosoft.com/

4 http://www.lynnon.com/

## Supplementary Material

Figure S1 Alignment of *ARC1* amino acid sequence.

Figure S2 Alignment of *SRK* amino acid sequences.

Figure S3 Expression analysis for SI-related genes.

Table S1 Primers used in SI-related gene analysis.

Table S2 The CI score and S-haplotype of F_2_ individuals; frequency histogram of F_2_ individuals.

Table S3 Information concerning QTL linkage markers and background markers.

Table S4 Information on each back-cross individual.

Table S5 Information on agronomic traits.
