# Supplementary figures and images for: Overcoming Cabbage Crossing Incompatibility by the Development and Application of Self-Compatibility-QTL- Specific Markers and Genome-Wide Background Analysis
Source: Front Plant Sci. 2019 Feb 26;10:189. doi: 10.3389/fpls.2019.00189 (PMC6399166; doi:10.3389/fpls.2019.00189)

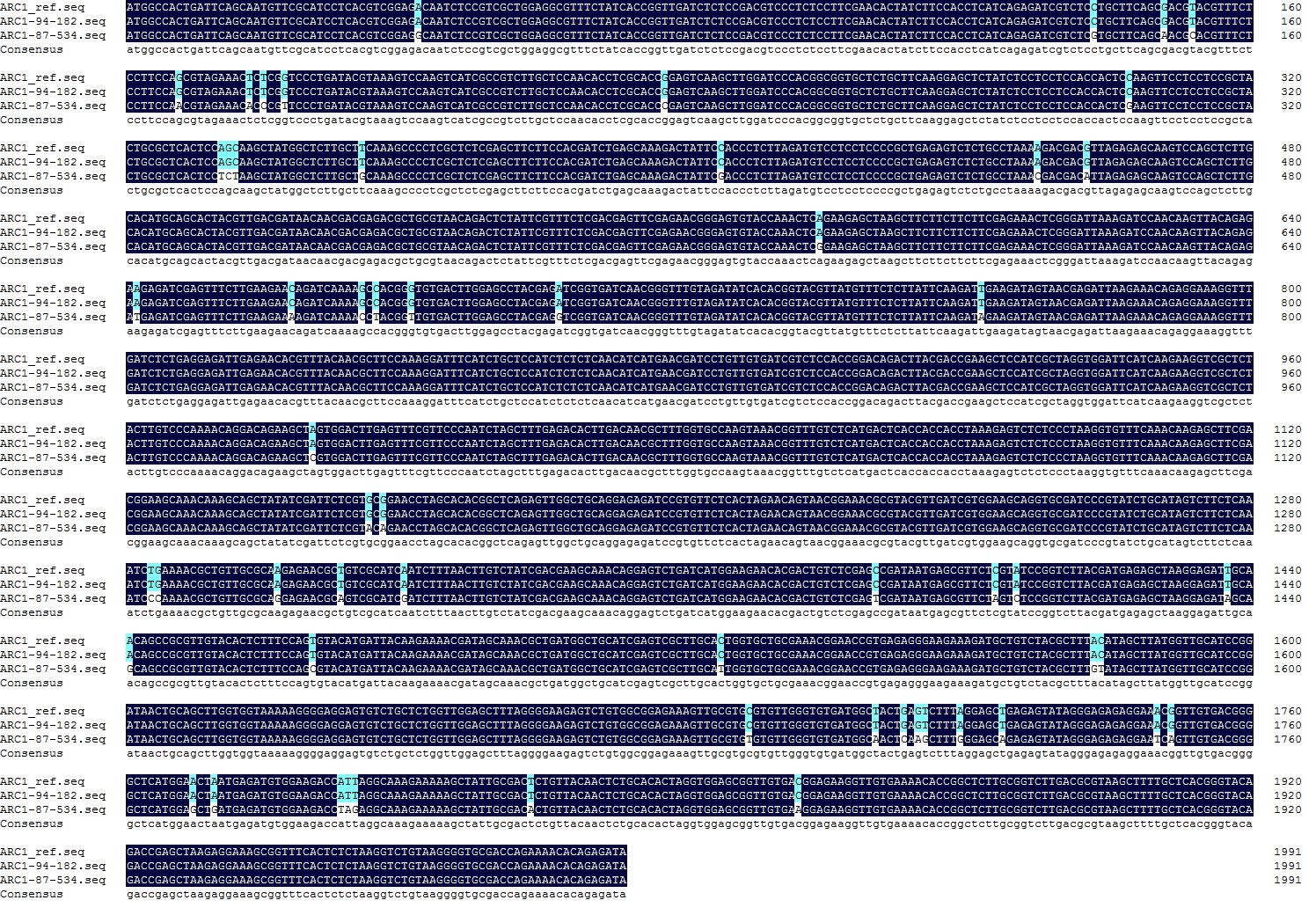

Supplement: Figure S1 — Alignment of ARC1 amino acid sequence. [file Image_1.JPEG]

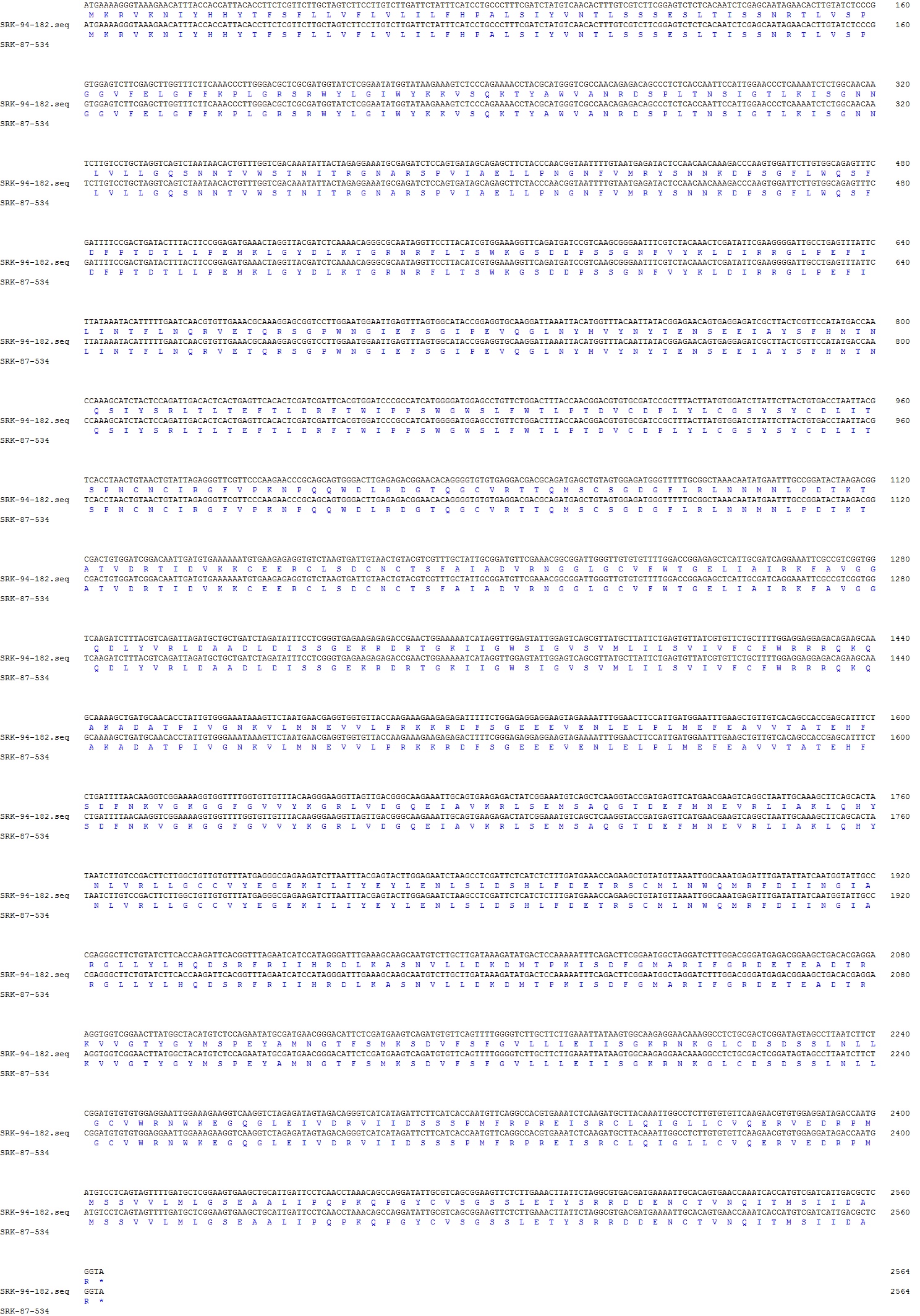

Supplement: Figure S2 — Alignment of SRK amino acid sequences. [file Image_2.JPEG]

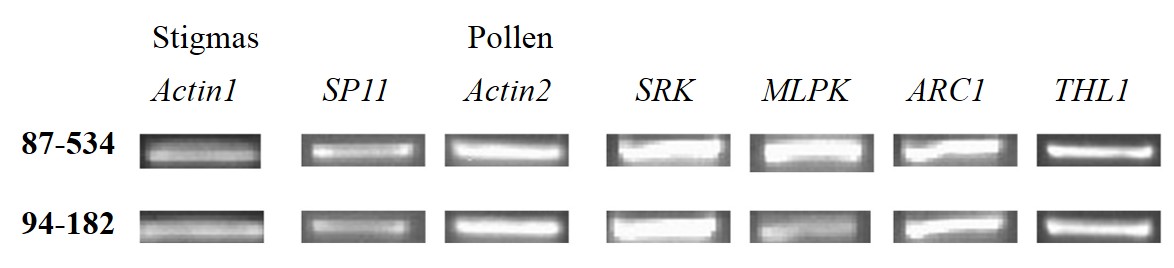

Supplement: Figure S3 — Expression analysis for SI-related genes. [file Image_3.JPEG]
